# SG-APSIC1036: Effect of quality improvement in medical devices preparation on increasing customers’ satisfaction in services of the Central Sterile Supply Department of Srinagarind Hospital

**DOI:** 10.1017/ash.2023.58

**Published:** 2023-03-16

**Authors:** Sasithorn Ruangprasertkul, Ponsawan Quobuwan

**Affiliations:** Srinagarind Hospital, Faculty of Medicine, Khon Kaen University, Khon Kaen, Thailand; Srinagarind Hospital, Faculty of Medicine, Khon Kaen University, Khon Kaen, Thailand

## Abstract

**Objectives:** Preparation of medical devices from central sterile supply department (CSSD) for use in hospital services requires quality and readiness for use. A guideline for good practice is necessary for safety, assurance, and maximum customer satisfaction, and to accommodate effective healthcare services. We sought to develop and improve medical-device preparation guidelines to satisfy clients. **Methods:** This action research was based on the concepts of Kaizen and eliminate–combine–rearrange–simplify (ECRS). The research was conducted in 3 phases. In the first phase, we designed the study, conducted problem analysis, and developed a plan for improving the preparation of medical devices. In the second phase, we improved the plan for implementation of medical-device preparation guidelines that the research team adapted and developed. We added inspection categories, trained staff members, conducted a focus group. We improved cleaning processes and the inventory system. In the third phase, we conducted an improvement evaluation for (1) quality improvement of medical device preparation and (2) client satisfaction. The research took place from January to December 2019. **Results:** The monthly percentages of medical equipment that passed quality criteria before and after the implementation plan were 91.82±1.19% and 95.33±1.25% (*P* ≤ .005). The average client satisfaction score increased from 76.80% to 83.40% (*P* = .006). **Conclusions:** The implementation of Kaizen and ECRS principles for quality improvement successfully increased the quality of equipment preparation and introduced standardized, quality guidelines. The plan–do–check–act (PDCA) process improved client satisfaction, staff performance, and operational efficiency while preventing damage to medical devices and improving readiness of use.

